# Genome Sequence of Bacteriophage GG32, Which Can Infect both *Salmonella enterica* Serovar Typhimurium and *Escherichia coli* O157:H7

**DOI:** 10.1128/genomeA.00802-16

**Published:** 2016-12-08

**Authors:** Su-Jin Chae, Taesoo Kwon, Sunjin Lee, Yeon Ho Kang, Gyung Tae Chung, Dae-Won Kim, Deog-Yong Lee

**Affiliations:** a Division of Enteric Diseases, Center for Infectious Diseases, National Institute of Health, KCDC, Heungdeok-gu, Cheongju-si, Chungcheongbuk-do, Republic of Korea; b Division of Biosafety Evaluation and Control, National Institute of Health, KCDC, Heungdeok-gu, Cheongju-si, Cheongbuk-do, Republic of Korea

## Abstract

We report here a new virulent *Salmonella enterica* serovar Typhimurium (*S*. Typhimurium) bacteriophage, GG32, which was isolated from the Guem River in the Republic of Korea. The strain can infect both *S*. Typhimurium and *Escherichia coli* (*E. coli*) O157:H7 and may be a good candidate for a bio-control agent.

## GENOME ANNOUNCEMENT

*Salmonella enterica* serovar Typhimurium (*S*. Typhimurium) and *Escherichia coli* (*E. coli*) O157:H7 are major food-borne pathogens causing serious diseases (1, 2). Bacteriophages have been used to bio-control agents in a variety of foods (3, 4). We report a new virulent bacteriophage GG32 isolated from the Guem River in the Republic of Korea. The strain had effective inhibition of both *S*. Typhimurium and *E. coli* O157:H7 by a single bacteriophage. Phage amplification and plaque purification were carried out by the agar overlay method using the host strain *S*. Typhimurium DT36. Morphological analysis showed that phage GG32 belongs to the family *Myoviridae*, it has an isometric head of 81 nm with a contractile tail approximately 125 nm long.

Phage was concentrated by the PEG 8000 precipitation and the genomic DNA was purified by the phenol extraction method (5). Sequencing library was constructed using the GS FLX Titanium rapid library preparation kit (Roche Diagnostics, Germany) according to the manufacturer’s instructions. The genome sequencing was performed using GS FLX platform and sequence reads were assembled using GS De Novo Assembler (version 2.9). Sequencing yielded a total of 33,207 reads (13,905,354 bp) with an average read length of 418.748 bp and average coverage of 88.5×. The GG32 genome consisted of 157,855 bp with a G+C content of 44.54%. The genome of GG32 showed approximately 56% nucleotide homology to *Salmonella* phage SFP10. The nucleotide homology of the genome was calculated using EMBOSS Stretcher (EMBOSS version 6.5.7.0) (6). Concordantly, *Salmonella* phage SFP10 was the closest neighbor of GG32 in whole-genome based phylogeny and both of those strains belong to viunalike virus clade (7). To calculate the phylogenetic distance, multiple sequence alignment was performed using MAFFT (version 7.221) (8) and RAxML (version 8.2.0) (9) with the Gamma GTR model used for the inference of the phylogenetic tree.

The genome was annotated using RAST (Rapid Annotations using Subsystems Technology) (10). The genome contained 195 predicted protein-coding sequences. Of those protein-coding sequences, 73 matched identified phage genes, including 24 genes involved in phage structure, 21 genes involved in DNA replication, 13 genes involved in phage physiology, nine genes involved in nucleotide biosynthesis, four regulatory genes, and two homing endonucleases. The remaining 141 protein-coding sequences encoded hypothetical proteins.

### Accession number(s).

The genome sequence of GG32 was deposited in DDBJ/EMBL/GenBank under the accession number KX245012.
